# Risk factors for HIV infection among female sex workers in Bangui, Central African Republic

**DOI:** 10.1371/journal.pone.0187654

**Published:** 2017-11-06

**Authors:** Jean De Dieu Longo, Marcel Mbeko Simaleko, Henri Saint-Calvaire Diemer, Gérard Grésenguet, Gilles Brücker, Laurent Belec

**Affiliations:** 1 Centre National de Référence des Maladies Sexuellement Transmissibles et de la Thérapie Antirétrovirale, Bangui, Central African Republic; 2 Unité de Recherches et d’Intervention sur les Maladies Sexuellement Transmissibles et le SIDA, Département de Santé Publique, Faculté des Sciences de la Santé de Bangui, Bangui, Central African Republic; 3 Université Paris Sud, Département de Santé Publique, Bicêtre Hospital, Assistance Publique-Hôpitaux de Paris, Le Kremlin-Bicêtre, France; 4 Direction Internationale, Assistance Publique-Hôpitaux de Paris, Paris, Franc; 5 Laboratoire de Microbiologie, hôpital Européen Georges Pompidou, Assistance Publique-Hôpitaux de Paris, Paris, France; 6 Faculté de Médecine Paris Descartes, Université Paris Descartes, Sorbonne Paris Cité, Paris, France; Centers for Disease Control and Prevention, UNITED STATES

## Abstract

**Objective:**

The aims of the study were i) to categorize female sex workers (FSW) according to socio-anthropologic criteria in Bangui; ii) to examine the association between a selection of demographic and risk variables with the different categories of female sex work as outcome, and iii) to investigate factors associated with HIV status.

**Methods:**

A cross-sectional questionnaire survey was conducted to describe the spectrum of commercial sex work in Bangui among 345 sexually active women. After collection of social and behavioral characteristics, each woman received a physical examination and a blood sample was taken for biological analyses, including HIV testing. The relationships between sociodemographic characteristics, behavioral variables involved in high risk for HIV as well as biological results were investigated by bivariate analysis in relationship with FSW categories as main outcomes, and by bivariate analysis followed by multivariate logistic regression analysis in relationship with HIV as the main outcome. The strength of statistical associations was measured by crude and adjusted Odds ratios (OR) and their 95% confidence intervals.

**Results:**

The typology of FSW comprised six different categories. Two groups were the “official” professional FSW primarily classified according to their locations of work [i) “*kata*“(18.55%) representing women working in poor neighborhoods of Bangui; ii) “*pupulenge”* (13.91%) working in hotels and night clubs to seek white men]. Four groups were “clandestine” nonprofessional FSW classified according to their reported main activity [i) “market and street vendors” (20.86%); ii) “schoolgirls or students” (19.13%) involved in occasional transactional sex (during holidays); iii) “housewives or unemployed women” (15.65%); iv) “civil servants” (11.88%) working as soldiers or in the public sector]. The overall prevalence of HIV-1 was 19.12% (66/345). HIV varied according to FSW categories. Thus, among professional FSW, the HIV prevalence was 6-fold higher in "*kata*" than "*pupulenge*" (39.13% *versus* 6.30%; *P* = 0.001). Among nonprofessional FSW, the "vendors" showed the highest HIV prevalence (31.91%), which was higher than in "students" (6.10%; *P* = 0.001), "civil servants" (9.83%; *P* = 0.005), and "housewives" (13.00%; *P* = 0.01). In bivariate analysis, the following variables showed statistically significant association with risk for HIV infection: nationality; age of first sexual intercourse; self-assessment of HIV risk; knowledge of HIV status; anal sex practice with last clients; irregular condom use in last week; consumption of alcohol; other psycho-active substances; past history of STIs; HBs Ag; HSV-2 and bacterial vaginosis. However, the variable “sex workers categories” dichotomized into professional *versus* nonprofessional FSW was no longer associated with HIV. In multivariate logistical regression analysis, HIV infection was strongly associated with nationality (15.65% *versus* 3.77%) [adjusted OR (aOR) 3.39: 95% CI:1.25–9.16, *P*<0.05]; age of first sexual intercourse (21.10% *versus* 14.00%) (aOR 2.13: 95% CI: 1.03–4.39, *P*<0.05); anal sex practice with last clients (43.40% *versus* 11.50%) (aOR 4.31: 95% CI:2.28–8.33, *P*<0.001); irregular condom use in past week (33.50% *versus* 3.00%) (aOR 5.49: 95% CI:1.89–15.98, *P*<0.001); alcohol consumption before sex (34.70% *versus* 7.80%) (aOR 2.69: 95% CI:1.22–4.96, *P*<0.05); past history of STIs (41.00% *versus* 10.80%) (aOR 2.46: 95% CI:1.22–4.97, *P*<0.05) and bacterial vaginosis (29.80% *versus* 4.29%) (aOR 6.36: 95% CI: 2.30–17.72, *P*<0.001).

**Conclusion:**

Our observations highlight the high level of vulnerability for HIV acquisition of both poor professional “*kata*” and nonprofessional “street vendor” FSW categories. These categories should be particularly taken into account when designing specific prevention programs for STIs/HIV control purposes.

## Introduction

The Central African Republic (CAR) is one of the countries in Central Africa the most affected by generalized epidemic of human immunodeficiency infection (HIV), with an overall prevalence in adult population of 4.9% in 2014 [1–3]. The principal mode of HIV transmission in the CAR is heterosexual [4]. The HIV epidemic in the CAR depicts a trend towards feminization, with an HIV prevalence among women (7.8%) double the prevalence among men (4.3%) in the same age group [1]. The dynamic of HIV epidemic in heterosexual population is particularly affected by the serial or concomitant occurrence of sexually transmitted infections (STIs) [5–7]. A strong synergistic association between heterosexual transmission and acquisition of HIV and genital herpes simplex virus type 2 (HSV-2) infection was demonstrated in sexually active adults [6,8]. Fighting the HIV epidemic in sub-Saharan Africa remains a major issue, especially in the CAR, a country where HIV epidemic was qualified as “*out of control*” [9]. In the present study, our aim was to better characterize the prevalence of HIV among female sex workers, a known high-risk group for HIV acquisition and transmission.

The population of female sex workers (FSW), according to the WHO terminology for commercial sex in women (WHO, 2014), includes young women who sell sex (YWSS) (less than 18 years) and adult FSW (more than 18 years). FSW an important group of HIV heterosexual transmission in the CAR, as in other sub-Saharan African countries [10–14]. Context-specific typologies of FSW are essential for the design of HIV intervention programmes [15]. However, little consideration has been given so far to the different patterns of female sex work in the CAR. In West Africa, Nagot and colleagues proposed a classification of commercial sex in Burkina Faso in six different categories, including four groups of non-professional FSW [16]. Most previous research studies focused on women who identified themselves as FSW. However, these represent only the visible side of the sex work network [17,18]. The nature and role of so-called “clandestine” sex work has not been properly characterized [16].

We carried out a formative sociologic and anthropologic survey in Bangui, the capital city of the CAR, in order to analyze the various characteristics and networks of female sex work and to propose a typology of the various categories of FSW [19]. The nature of “clandestine” sex work which has been the matter of little attention so far, especially in Central Africa, was particularly considered. This situation analysis yielded important novel information regarding the sex work network in Bangui. Female sex work appeared remarkably heterogeneous. Besides the true professional FSW, “clandestine” nonprofessional FSW could also be identified. Finally, the broad spectrum of FSW was grouped into 6 principal categories according to their own social and behavioral characteristics. Classification of the different YWSS and FSW operating in the CAR may be a tool to assess the dynamics of HIV epidemic and to design and adapt operational intervention programs against HIV infection and other STIs [16, 17, 20, 21–25]. Furthermore, specific and adapted interventions may help to propose antiretroviral treatment in HIV-infected FSW with potential impact on decreasing sexual transmission of HIV [24, 26].

We carried out a cross-sectional study to estimate, the prevalence and factors of HIV infection in Bangui, according to the various categories of FSW (YWSS and adult FSW) previously identified during the anthropologic survey. The goal was to propose specific interventions targeting FSW according to each socio-behavioral category. Finally, we examined the association between a selection of demographic and risk variables with the different categories of female sex work as outcome, and investigated factors associated with HIV status.

## Material and methods

### Study design

We conducted a cross-sectional survey of 345 females involved in commercial sex transactions living in Bangui and attending the *“Centre National de Référence des Maladies Sexuellement Transmissibles et du SIDA”* (*CNRMST/SIDA*) to evaluate their principal socio-demographic and behavioral characteristics in relationship with their HIV status. The *CNRMST/SIDA* of Bangui is the main clinic center of the capital city for voluntary HIV counseling and testing and for diagnosing and caring STIs [7]. The study was a population-based cross-sectional survey in 2014, using a face-to-face questionnaire to collect data among YWSS and adult FSW populations living in Bangui. These women were selected and characterized during the previous anthropologic and sociologic survey conducted in 2013 [19].

### Study population

The study population included sexually active women in well-known areas of sexual transactions in Bangui, having more than 2 sexual partners (other than their regular partner) during the last 3 months and reporting having received money or “gifts” in return of sexual relationships.

### Recruitment procedure

The study was carried out in 8 locations in Bangui, including 4 major dancing venues and 4 other locations notoriously for sexual activity, such as the French military area near the airport. In addition, 13 institutions of learning, including secondary schools and universities, were also selected. All venues listed in the sociologic and anthropologic survey were selected for the cross-sectional study. In each inclusion area, women were recruited voluntarily and independently of their age participation

Over a three month period, 12 peer educators reached out prospectively to recruit all women attending the 21 selected locations. The purpose of the study was explained verbally. After oral consent, the selected women were invited to attend the in-person interviews. Transportation allowance was provided to attend the *CNRMST/SIDA* of Bangui. Additional benefits for all women included free HIV testing, gynecological examination for STIs and laboratory analysis if necessary, and appropriate treatment for those suffering from genital infections or HIV infection. Importantly, the invitation to attend the clinic center was proposed to all women, regardless of possible paid sexual practices. Women who did not give oral consent were excluded, as well as those who had had sexual relationships to obtain a job or to have good results in classroom.

### Laboratory procedures

Each volunteer underwent several biological analyses. Screening for HIV infection was carried out according to the sequential algorithm combining Alere Determine HIV-1/2 test (Alere Medical Co. Ltd., Matsudo-shi, Chiba-ken, Japan), and Uni-Gold™ HIV (Trinity Biotech Manufacturing Ltd., Bray, Ireland). In case of indeterminate results, the sample was subjected to additional HIV testing (Genscreen™ ULTRA HIV Ag-Ab, Bio-Rad, Marnes-La-Coquette, France). Hepatitis B surface antigen (HBs Ag) was detected by Monalisa HBs Ag (Bio-Rad). Syphilis serology used nontreponemal RPR test (Carbon, Cypress Diagnostics, Belgium) and treponemal TPHA test (Syphagen, Biokit, Barcelona, Spain). Herpes simplex virus type 2-gD-specific serology was carried out by the HSV-2 BioElisa kit (Biokit), as described [8]. Bacterial vaginosis was detected by Gram staining of fresh vaginal swabs using the Nugent score, as previously described [27].

### Study outcomes

All attending women benefitted from the free clinic services, and completed a face-to-face questionnaire. The questionnaires were administered at the *CNRMST/SIDA* by experienced counselors, specifically trained for this purpose. The questionnaire contained demographic and behavioral items, knowledge of HIV infection and associated diseases and comorbidities, and questions on possible sexual transactions. In detail, the demographic and risk variables (n = 23) taken into consideration to assess the relationship between FSW and HIV serology were as follows:

✓ *Sociodemographic variables* included: age, education level (divided into three levels as follows: no formal education, primary/secondary education and higher education), occupation (as the principal source of income), nationality, matrimonial status (divided in unmarried or married), number of children;
✓ *Sexual risk variables* for STI and HIV/AIDS acquisition or transmission included: age of first sexual intercourse (before or more than 16 years); basic knowledge on HIV infection (volunteers were asked whether they have ever heard of HIV/AIDS, and whether they are able to name at least one of the classical individual prevention measures against HIV acquisition, *e*.*g*. abstinence, faithfulness and reporting consistent condom use), condom use (FSW spontaneously reporting condom use as effective tool to avoid STI and HIV/AIDS were considered at low risk, while those not reporting consistent condom use during the last 3 months or during the last week before the questionnaire were at high risk for HIV and STI acquisition), participation in prevention intervention (including FSW attending voluntary counselling and testing for HIV and high perception of risky sexual behaviors associated with consistent condom use with their clients), duration of commercial sex work and past history of STI (genital ulcer or vaginal discharge), practice of anal sex, consumption of alcohol before sex work as well as consumption of other psycho-active substances including cannabis, glues inhalation and methamphetamines;
✓ *Sexually transmitted infections*, diagnosed by physical and clinical examination and laboratory investigations.

### Statistical analyses

The questionnaire data were entered into an Excel sheet and analyzed using Epi Info^TM^ version 3.5.1 (Center for Disease Control and Prevention, Atlanta, Ga, USA). P<0.05 was considered as statistically significant. The strength of statistical associations was measured by crude and adjusted Odds ratios (OR) and their 95% confidence intervals.

The sex worker categories were grouped into professional and nonprofessional FSW categories for statistical analyses of factors associated with HIV.

In a first analysis, FSW categories were used as main outcomes, in order to investigate by bivariate analysis the relationships between sociodemographic characteristics, behavioral variables involved in high risk for HIV as well as biological results among the 345 included women practicing paid sex. The Chi-2 test was used to compare the various proportions between categories. For the comparison of sociodemographic and behavioral characteristics between FSW categories, the category of baseline reference was taken as that of “vendor” because it contained the highest number of participants.

In a second analysis, HIV infection constituted the main outcome; then, the study variables taken separately were firstly analyzed by bivariate analysis, in order to determine the explanatory variables possibly associated with HIV infection, *i*.*e*. showing a crude Odds ratio (OR) with P <0.05. The 14 significant variables resulting from initial bivariate analysis were then included in a multivariate logistic regression model in order to adjust for possible confounders. The proportion of missing data for the main variable of interest (*i*.*e*. HIV) was considered as negligible because it was extremely low (2.5%) corresponding to the 9 participants lost to follow up. HIV testing could not be carried out on these 9 participants.

### Ethics statement

The study was formally reviewed and approved by the Scientific Committee Faculty of Health Sciences of Bangui (“Comité Scientifique Chargé de la Validation des Protocoles d’Etudes et des Résultats”/”CSCVPER”) (agreement UB/FACSS/CSCVPER), which serves as the National Ethical Review Committee. All participating women gave their informed oral consent to participate in the study. No consent from the parents or guardians of minor women could be obtained. For each included woman, the record of the consent to participate in the study was documented on the respective questionnaire. This consent procedure was formally approved by the National Ethical Committee.

## Results

A total of 345 FSW questionnaires were included in the analysis, including 93 YWSS and 252 FSW.

### Participant’s characteristics

In 2013, 2,512 women received education about the objectives of the study, in addition to advice on sexual and reproductive health, as previously reported [19]. Among the sensitized women, 1,384 women attended the *CNRMST/SIDA*, and 357 (25.70%) of them reported gainful compensation for sex within the last 3 months and having at least 3 sexual partners during the 3 past months. However, 12 participants were excluded, including 3 with incomplete questionnaires and 9 were lost to follow up. Finally, 345 FSW participants with complete questionnaires and biological analyses were included in the analytic sample. Among them, 112 (32.50%) women reported regular paid sex transactions (commercial) as their main source of income, identified themselves as FSW, and were classified as “professional” FSW. The remaining 233 (77.50%) women reported another activity as their main source of income (n = 167) or were still schoolgirls/students (n = 66), but nevertheless entertained commercial sex transactions over a recent period (last 3 months) with more than 2 sexual partners apart from their regular partner, and thus were classified as “nonprofessional” FSW.

### Sociodemographic, behavioral and biological characteristics according to FSW categories

According to socio-behavioral characteristics, FSW were classified in 6 different categories as depicted in Table 1. Firstly, the “official” professional FSW (32.50%, n = 112) were primarily classified according to their site of work. Indeed, there was a marked separation between two categories of professional FSW, depending on whether they work downtown, near hotels, in bars and night clubs, or in peripheral areas of Bangui. The first category of professional FSW (13.90%, n = 48) included the “*pupulenge*” a Sango (the national language) word meaning *dragonfly* (also called “*gba moundjou*”, meaning literally “woman who likes to have sex with White”). This represents a Black lady who has sex with White men (*i*.*e*. high class FSW), and travels around the city from hotels to night clubs, looking for wealthy clients, with a preference for French men. The second category of professional FSW included women working in poor neighborhoods of Bangui, called “*kata*” (a pejorative word with no other Sango meaning, dedicated to whores) (18.60%, n = 64). Secondly, the nonprofessional FSW (67.50%, n = 233) were classified in 4 categories according to their reported main activity (“street vendors”, “schoolgirls or students”, “housewives” or “unskilled civil servants”). The first category of nonprofessional FSW was “market and street vendors” (20.80%, n = 72) consisting of mobile women selling fruits or vegetables along the main roads of the city by means of a tray carried on their head. The second category was “schoolgirls or students” (19.10%, n = 66) involved in occasional transactional sex, particularly during holydays. Most of them do so to pay school fees or for their living expenses, but others just want money to buy fashion clothes or jewelery. The third category of nonprofessional FSW was “housewives or unemployed women” (15.70%, n = 54). Finally the fourth category of nonprofessional FSW was “unskilled female civil servants” (11.90%, n = 41), working as soldiers or in the public service. Taken together, besides the professional FSW, non-professional FSW appeared clearly as an extremely frequent second population of FSW.

**Table 1:** Categories of female sex workers (FSW), principal sociodemographic characteristics and variables involved in high risk for HIV and biological results among 345 women practicing paid sex and living in Bangui.

	Professional FSW [112 (32.5)]*		Nonprofessional FSW [233 (67.5)]				Total
	*Kata* [64 (57.10%)]*	*Pupulenge* [48 (42.90%)]	*Vendors* [72 (30.90%)]	*Housewives* [54 (23.20%)]	*Civils servants* [41 (17.60%)]	*Students* [66 (28.30%)]	
**Age**							
Median (years)	20	21	25	27	27	20	23
Range	15–36	16–29	15–38	18–46	22–47	16–28	15–47
≤18 years old (%)	14	21	14	2	0	17	11.90
**Age of first sexual intercourse** (%)^μ^							
< 16 years	67.20	56.30	54.20	50.00	31.70	39.40	50.70
16 years and more	32.80	43.80	45.80	50.00	68.30	60.60	49.30
**Number children**							
3 or more	1.56	2.08	30.55	55.55	34.15	0.00	19.71
1–2	29.64	52.08	48.61	37.04	56.09	33.33	41.74
0	68.80	45.84	20.84	7.41	9.86	63.67	38.55
**Marital status**							
Unmarried	100.00	94.00	82.00	41.00	49.00	82.00	75.65
Married	0.00	6.00	18.00	59.00	51.00	18.00	24.35
**Education level** (%)^**β**^							
Illiterate	58.00	0.00	43.87	3.07	0.00	0.00	15.07
Secondary level	36.00	81.00	43.00	50.00	2.00	50.00	68.99
Superior level	6.00	19.00	13.30	38.90	98.00	50.00	15.94
**Nationality**							
Central Africa Republic	81.00	78.00	94.00	94.00	94.00	100.00	91.10
Congo Democratic Republic	13.00	13.00	3.00	3.00	4.00	0.00	5.80
Others	6.00	9.00	3.00	3.00	2.00	0.00	4.10
**Knowledge on HIV/AIDS**^**∑**^							
Weak	6.00	22.00	63.00	2.00	17.00	5.00	21.50
Middle	17.00	33.00	25.00	7.00	17.00	7.00	18.50
Satisfactory	77.00	45.00	12.00	91.00	66.00	88.00	60.00
**Self-assessment of HIV risk in future**							
No	37.50	83.33	16.67	55.56	31.70	53.03	44.64
Yes	62.50	16.67	83.33	44.44	68.30	46.97	55.36
**Duration of prostitution** (%)^**β**^							
≤1 year	20.11	6.20					19.00
2–5 years	67.79	58.00	NA	NA	NA	NA	59.00
>5 years	12.10	35.80					22.00
**Past history of STIs** (%)^**£**^							
Yes	37.50	18.79	43.10	27.80	14.62	15.20	36.50
No	62.50	81.21	56.90	72.20	84.38	84.80	63.50
**Anal sex with last clients** (%)^μ^							
Yes	32.80	25.00	31.90	24.10	12.19	13.51	24.00
No	67.20	75.00	68.10	75.90	87.81	86.49	76.00
**Condom use in last 3 months** (%)^**£**^							
No or rarely	93.81	25.00	84.70	74.10	43.90	45.49	64.00
Always	6.29	75.00	15.30	25.90	56.10	54.51	36.00
**Alcohol consumption** (%)^**£**^							
No or rarely	12.53	89.59	51.44	50.00	80.59	75.82	57.44
Every day	87.50	10.41	48.63	50.00	19.51	24.21	42.63
**HSV-2 serology**							
Positive	12.50	4.30	15.30	13.60	14.60	13.60	11.88
Negative	87.50	95.70	84.70	86.70	85.40	86.40	88.11
**HBs Ag detection**							
Positive	67.20	22.90	11.11	11.12	12.20	4.50	22.03
Negative	32.20	77.10	88.89	88.88	87.80	95.50	77.97
**Syphilis serology**							
Positive	7.80	0.00	4.20	3.70	0.00	0.00	3.48
Negative	92.20	0.00	95.80	96.30	0.00	0.00	96.52
**Bacterial vaginosis**							
Positive	90.60	62.50	70.80	25.90	24.40	63.80	59.42
Negative	9.40	37.50	29.20	74.10	75.60	36.20	40.58
**HIV serology** (%)^μ^	39.13	6.30	31.91	13.00	9.83	6.10	19.12
**HIV prevention Odd ratio** (%)^**Ω**^	0.73	7.04	1	3.15	4.29	7.17	-
**95% confidence interval**	[0.36–1.48]	[2.11–30.82]	NA	[1.25–8.50]	[1.44–15.61]	[2.46–25.69]	-

^*^ [n (%)], n: Number and percentage in parentheses

^β^ Not significant (*P* ˃ .05, by Chi-_2_ test:)

^μ^ < .05. by Chi-_2_ test

^£^ < .001. by Chi-_2_ test

^**Ω**^For the comparison of sociodemographic and behavioral characteristics between FSW categories, the category of baseline reference was arbitrarily taken as that of “seller” because its largest number of participants

^**∑**^: The knowledge about HIV / AIDS transmission is based on the knowledge of the three main modes of prevention: (i) limitation of sexual intercourse to a single faithful uninfected partner; (ii) use of the condom at each sexual intercourse; and (iii) sexual abstinence and rejection of erroneous ideas, as assessed in the National Institut Centrafricain des Statistiques et des Etudes Economiques et Sociales (2006) for categorization of HIV knowledge [28]. The scale of knowledge on HIV was defined as follows: Weak knowledge: no right answer; Middle knowledge: one of 3 right answers; Satisfactory knowledge: 2 or 3 of 3 right answers.

NA: Not attributable; STIs: Sexually transmitted infections.

Professional FSW [median age of 21 years (range, 15–36 years)] were younger than nonprofessional FSW [median age 25 (range, 15–47 years)] (*P*<0.05); 30% of professional FSW were less than 18 years against 15% of nonprofessional FSW (*P*<0.05). The proportion of foreigners was 3-times higher among nonprofessional FSW than among professional FSW (17.10% *versus* 5.70%; *P*<0.05). Professional FSW showed slightly higher levels of education than nonprofessional FSW, achieving a level of secondary education of 55% *versus* 38% (*P*<0.05). Among professional FSW, the group of “*kata*” were slightly younger than “*pupulenge*” (*P*<0.05). The level of education was significantly lower among “*kata*” (*P*<0.001). Nonprofessional FSW practiced occasional paid sex as secondary source of income and did not consider and report themselves as FSW. They were most often originating from the CAR. “Schoolgirls and students” were the youngest, with a median age of 20 years, and the “housewives” and “civil servants” the oldest, with a median age of 27 years (*P*<0.01). The level of education among the 4 subgroups was heterogeneous, with “civil servants” showing generally a higher level, followed by “schoolgirls/students” and by “housewives”; “street vendors” were the least educated subgroup, with nearly half of them being illiterate (*P*<0.05). Most “housewives” and “civil servants” were married, while most “street vendors” and “schoolgirls/students” were single (*P*<0.01).

The median age of first sexual intercourse was younger in professional [median age of 16 years (range, 10–19 years)] than in nonprofessional [median age of 17 years (range, 11–24 years)] FSW (*P*<0.01); and the proportion of women reporting a sexual experience before the age of 15 was higher in professional (63%) than nonprofessional (47%) FSW (*P* <0.01). The duration of sex work does not differ dramatically between categories. Past history of STIs was noticed in 30% of professional FSW and 27% of nonprofessional FSW (NS). However, among nonprofessional FSW, past history of STIs was noticed more frequently in “street vendors” and in one-third of “housewives” (*P*<0.05). HBs Ag detection was more frequent in professional FSW [54/112 (48.20%); “*kata*”, 43/64 (67.20%); “*pupulenge*”, 11/48 (22.90%)] than in nonprofessional FSW [22/233 (9.40%); “civil servant”, 5/41 (12.20%); “street vendors”, 8/72 (11.11%); “housewives”, 6/54 (11.12%); “students”, 3/66 (4.50%)] (*P*<0.0001). The proportion of women who proposed a condom to their male sexual partner during the last sexual intercourse, and the proportion of those who effectively used a condom during the last sexual intercourse were similar in professional and nonprofessional FSW, 17% *versus* 19% and 57% *versus* 64%, respectively (NS). The proportion of women who proposed a condom to their male sexual partner during the last 3 months, and the proportion of those who effectively used a condom during the last sexual intercourse were much higher among “*pupulenge*” than “*kata*” [75% *versus* 6% (*P*<0.001), and 100% *versus* 38% (*P*<0.001)]. Most “schoolgirls/students” and “civil servants” used condoms regularly, while only a minority of “housewives” and even lesser number of “street vendors” used them (*P*<0.005). Anal sex with last clients was frequent among study FSW, higher among professional FSW than among nonprofessional FSW [(29.50% *versus* 21.50% (*P*<0.05)], higher among “*kata*” than “*pupulenge*” [33% *versus* 25% (*P*<0.005)], and higher among “sellers” and “housewives” than “civil servants” and “students” [32% *versus* 24.1% (*P*<0.005)]. The percentage of women reporting regular (daily) consumption of alcohol was higher in official FSW (55% consuming more than 3 beer bottles of 65 cl) than “clandestine” FSW (37% consuming more than 3 beers bottles of 65 cl) (*P*<0.002). The “*kata*” consumed more alcohol (and more psycho-active substances, not shown) than the “*pupulenge*” (*P*<0.001). Alcohol was more consumed by around half of “housewives” and “street vendors” and one-quarter of “schoolgirls/students” and “civil servants” (*P*<0.01).

In 27% of cases, FSW were YWSS of less than 18 years. The majority of FSW originated from the CAR (91.10%) and, to a lesser extent, from other neighboring countries, including Democratic Republic of Congo (5.80%) and other countries (4.10%) (Democratic Republic of Congo, Chad and Cameroon). Most FSW (68.99%) showed secondary level education, equally distributed between college and high school; a minority (15.94%) had high education level, whereas the remaining (15.07%) had low or very low education level.

Finally, HIV infection prevalence varied according to FSW categories (Table 1). There was a trend of higher HIV prevalence among professional than nonprofessional FSW (22.30% versus 16.30%, respectively), but the difference was not statistically significant (P = 0.055). Among professional FSW, the prevalence of HIV infection was much higher among “*kata*” than “*pupulenge*“(39.13% *versus* 6.30%) (P<0.001). Among nonprofessional FSW, “students”, “civil servants” and “housewives” were less often infected (6.10%, 9.83% and 13.00%, respectively), whereas “sellers” showed very high HIV prevalence (31.91%).This large difference in HIV infection between categories was confirmed by highly significant Chi-_2_ test for trend (P<0.001).

### Sociodemographic, behavioral and biological characteristics of FSW and relationship with HIV infection by univariate and logistic regression analysis

None of the included FSW missed testing for HIV and STI. In the study population of 345 FSW, the prevalences of HIV-1, active hepatitis B (positivity for HBs Ag), circulating IgG antibodies to HSV-2 and active syphilis, were 19.12% (n = 66), 22.03% (n = 76), 11.88% (n = 41) and 3.48% (n = 11), respectively.

Concerning the sexual behavior of the FSW population (Table 2), HIV positivity was significantly associated with anal sex with last clients (practice of anal sex: 43.40% HIV^+^ *versus* only 11.5% HIV^+^ when exclusive vaginal intercourse), the lack systematic use of condom use in last 3 months (29.40% *versus* 0.80%) and in last week (33.50% *versus* 3.0%). There was no relationship between the duration of sex work and HIV status. HBs Ag detection was more frequent in FSW practicing anal sex [29/54 (34.90%) *versus* 47/215 (17.90%); *P*<0.002)].

**Table 2:** Bivariate and multivariate analyses using socio-behavioral characteristics and biological results variables in association with HIV status among 345 female sex workers.

Characteristic	HIV infection	Bivariate analysis	Multivariate Analysis^μ^
	Positive [n (%)]	Crude OR [95% IC]	Adjusted OR [95% IC]
**Age** (years)			
≤ 19	22 (23.91)	1	
20–24	21 (18.26)	1.41 [0.72–2.70] *	-
25–29	16 (16.49)	1.59 [0.78–3.27] *	-
≥ 30	7 (17.07)	1.53 [0.59–3.92] *	-
**Educational level**			
Never went to school	15 (28.85)	1	-
Secondary/ Higher education	53 (26.45)	1.84 [0.94–3.59] *	-
**Nationality**			
Central Africa Republic	54 (15.65)	1	
Others	13 (3.77)	0.8 0 [0.44–1.60] **	3.39 [1.25–9.16] **
**Marital statut**			
Unmarried	54 (15.65)	1	
Married	13 (3.77)	1.27 [0.65–2.47] *	-
**Number Children’s**			
More than 3	13 (18.60)	1	
1–2	25 (17.45)	0.91 [0.4–1.93] *	-
0	29 (22.15)	1.25 [0.60–2.59] *	
**Age of first sexual intercourse**			
15 years or less	42 (21.10)	1	
16 years or more	24 (14.00)	1.95 [1.12–3.39] **	2.13 [1.03–4.39] **
**Knowledge on HIV/AIDS**			
Weak	12 (47.40)	1	
Middle	15 (23.30)	1.51 [0.62–3.69] *	NS****
Satisfactory	39 (16.00)	2.41 [1.12–5.19] **	
**Self-assessment of HIV risk in future**			
No	17 (11.00)	1	
Yes	49 (25.70)	0.36 [0.20–0.66] ***	NS
**Knowledge on HIV serostatus**			
No	51 (28.00)	1	
Yes	15 (9.30)	1.93 [3.75–7.41] ***	NS
**Sex anal with last client**			
Yes	36 (43.40)	1	1
No	30 (11.50)	5.87 [3.16–11.01] ***	4.31 [2.28–8.33] ***
**Condom use in last week**			
No or rarely	61 (33.50)	1	1
Always	5 (3.00)	15.89 [5.85–46.47] ***	5.49 [1.89–15.98] ***
**Sex workers categories**			
**Professional FSW**			-
*Kata*	64 (57.10)	9.88 [2.92–36.61]***	-
*Pupulengue*	48 (42.90)	1.00 [0.13–5.80]***	-
**Nonprofessional FSW**			-
Vendors	72 (30.90)	7.22 [2.13–26.69]***	-
Housewives	54 (23.20)	2.31 [0.53–10.01]***	-
Civil servants	41 (17.60)	1.64 [0.33–8.64]***	-
Students	66 (28.30)	-	-
**Duration of prostitution**^**Ω**^			
>5 years	5 (18.50)	1	-
2–5 years	17 (31.50)	0.49 [0.14–1.70] *	-
<2 years	7 (22.60)	0.78 [0.18–3.30] *	-
**Alcohol consumption**			
Every day	51 (34.70)	1	1
No or fold per week	15 (7.80)	3.17 [6.22–12.29] ***	2.69 [1.22–4.96] **
**Cannabis consumption**			
Every day	9 (81.10)	1	
No or fold per week	57 (17.00)	4.19 [21.82–151.01] ***	NS
**Others drugs**^**£**^ **consumption**			
Every day	22 (45.80)	1	
No or fold per week	44 14.80)	2.44 [4.76–9.81] ***	NS
**Past history of STIs**			
Yes	39 (41.00)	1	1
No	27 (10.80)	3.09 [5.88–10.59] ***	2.46 [1.22–4.96] **
**HBs Ag detection**			
Positive	33 (3.40)	1	
Negative	34 (12.62)	5.32 [2.78–9.77] ***	NS
**HSV-2**			
Positive	15 (36.57)	1	
Negative	51 (16.78)	2.83 [1.32–6.03] ***	NS
**Bacterial vaginosis**			
Positive	61(29.80)	1	1
Negative	6 (4.29)	9.53 [3.81–25.04] ***	6.36 [2.30–17.72] ***

^μ^ There was no missing data in multivariate logistic regression analysis.

* > .05 by Chi-_2_ test

** < .05 by Chi-_2_ test

*** < .001 by Chi-_2_ test

**** NS = Not significant, *i*.*e*. > .05 by Chi-_2_ test

^Ω^ Non attributable for nonprofessional female sex workers (n = 112)

^£^ Tramadol is an opioid pain medication used to treat moderate to moderately severe pain.

HBs Ag: HBs antigen of hepatitis B virus; HSV: Herpes simplex virus; CI: Confidence interval; OR: Odds ratio; STIs: Sexually transmitted infections

Concerning the use of alcohol and other psycho-active substances (Table 2), HIV positivity was significantly associated with regular consumption of alcohol (34.70% *versus* 7.80%), cannabis (81.10% *versus* 17.0%), glue (41.70% *versus* 18.30%) and tramadol (45.80% *versus* 14.80%).

Concerning the other remaining variables including past history of STIs, hepatitis B virus infection, HSV-2 and bacterial vaginosis in FSW population (Table 2), HIV positivity was significantly with past history of STIs (41.0% *versus* 10.80%), positivity of syphilis (63.60% *versus* 17.70%) and HSV-2 (43.40% *versus* 12.60%) serologies, active hepatitis B virus infection (HBs Ag^+^) (36.57% *versus* 16.80%) and the presence of bacterial vaginosis (29.80% *versus* 4.29%).

Interestingly, the prevalence of FSW co-infected by HIV and hepatitis B virus was unexpectedly high [33/76 (43.40%)]. In bivariate analysis, the following variables were significantly associated with HIV infection: nationality, age of first sexual intercourse, self-assessment of HIV risk, knowledge of HIV status, anal sex practice with last clients, irregular condom use in last 3 last week, consumption of alcohol or other psycho-active substances, past history of STIs, HBs Ag, HSV-2 and bacterial vaginosis. The variable “sex workers categories” was not significantly associated with HIV. In multivariate logistic regression analysis using the variables shown as significant in bivariate analysis, HIV infection in FSW populations was strongly associated with 7 variables depicted in the Table 2, including nationality adjusted OR (aOR) 3.39: 95% CI:1.25–9.16, *P*<0.05), age of first sexual intercourse (aOR 2.13: 95% CI:1.03–4.39, *P*<0.05), anal sex practice with last clients (aOR 4.31: 95% CI:2.28–8.33, *P*<0.001), irregular condom use in last week (aOR 5.49: 95% CI:1.89–15.98, *P*<0.001), alcohol consumption before sexual activity (aOR 2.69: 95% CI:1.22–4.76, *P*<0.05), past history of STIs (aOR 2.46: 95% CI:1.22–4.97, *P*<0.05) and bacterial vaginosis (aOR 6.36: 95% CI:2.30–17.72, *P*<0.001).

## Discussion

In the present study, we used a unique typology of six different FSW categories among women involved in commercial sex transactions living in Bangui, divided into “official” professional FSW and “clandestine” nonprofessional FSW, thus demonstrating a very heterogeneous population. The overall HIV-1 prevalence of HIV-1 among study FSW was particularly high, reaching nearly 20%, highlighting vulnerable and high risk behavior regarding HIV infection and other STIs. Remarkably, the HIV prevalence varies differentially according to FSW categories, from 6.1% to 39.1%. Taken together, the proposed typology for commercial female sex work in Bangui may be useful to propose intervention programs against HIV and other STI adapted and specific to the broad spectrum of FSW in the Central African Republic.

### Characteristics of study FSW population

In the present study, we addressed HIV testing in a sub-group of FSW attending the *CNRMST/SIDA* of Bangui. The high rate of HIV testing acceptance (97%) is likely explained by the high quality of counselling services and management of STIs and HIV infection at the *CNRMST/SIDA*, as previously reported [7]. Note that the *CNRMST/SIDA* also provides care packages for men who have sex with men [29] as well as for women victims of sexual violence.

Half (51%) of FSW reported past history of STIs, a finding which was strongly associated with HIV infection. This observation is reminiscent of numerous previous reports demonstrating that STIs act as a major cofactor of HIV acquisition in sub-Saharan Africa [30]. The strong association between HIV infection and STIs is also well known in the CAR [5]. The difficulties encountered by FSW in accessing health facilities and adequate information on HIV and STIs expose a vicious cycle of HIV and STIs, and favor the risk of STIs with further HIV acquisition [31].

In our series, the vast majority of FSW were using condoms (84%). Participating FSW who do not consistently use condoms during sex with clients showed increased risk of HIV infection. On the other hand, previous observations in low-income countries showed that condom use in FSW greatly reduced the expected monetary compensation, translating into financial losses [32–34].

One fifth (19%) of our study FSW reported practicing anal sex with their last clients. This observation is particularly relevant regarding further risk of HIV acquisition. Indeed, it is well known that risk of HIV acquisition and transmission during anal sex is significantly higher than during vaginal intercourse [14, 35]. Thus, receptive anal intercourse in study FSW was associated with increased risk of acquiring HIV by a factor of 2.4 (range, 1.3–4.9), by comparison with those practicing only vaginal intercourse. Anal sex practice has been commonly reported among sex workers in sub-Saharan Africa [36–39]. Thus, ignoring anal intercourse in HIV prevention interventions will miss important opportunities to prevent HIV acquisition [40].

Study FSW who consumed alcohol before or during sex work showed increased risk of HIV infection. The association between alcohol abuse and unprotected sex is well documented [41–43], and alcohol may be considered as a risk factor *per se* for sexual transmission of HIV and other associated STIs [21,44].

In univariate analysis, positive syphilis and HSV-2 serologies, HBs Ag detection and the diagnosis of bacterial vaginosis were associated with HIV infection in study FSW. However, the association was not retained in multivariate analysis, likely because of insufficient number of enrolled participants. High rates of positive syphilis and HSV-2 serologies were previously reported in FSW [11,22,45–49], and are considered as classical risk factors for HIV acquisition in sub-Saharan Africa [5,6,10,17,30,50]. Bacterial vaginosis was frequent in study FSW. It is well known that bacterial vaginosis may be associated with unprotected sex [51]. The evidence that bacterial vaginosis predisposes women to higher risk for HIV infection is well documented [52–54]. Finally, HSV-2 infection and bacterial vaginosis may contribute independently to HIV acquisition [55]. These findings confirm the high vulnerability of young African women regarding to STIs and bacterial vaginosis and emphasize the need for specific control interventions. These should include affordable and user-friendly services.

The CAR is endemic for hepatitis B virus infection [56, 57]. In our study, we used HBs Ag as a marker of hepatitis B virus infection contagiousness: the overall prevalence of HBs Ag among FSW in Bangui was 22.0%; the professional FSW were more likely to be infected by hepatitis B virus than nonprofessional FSW; the HBs Ag prevalence in HIV-positive FSW reached 43.4%. The prevalence of hepatitis B infection among study FSW was higher than those previously reported in young sexually active adults (14.0%) [56] or students (15.5%) [57] living in Bangui. Interestingly, the prevalence of hepatitis B virus infection among study FSW was higher in FSW practicing anal sex, which constitutes well-documented risk factor for hepatitis B virus acquisition [58].None of them were injecting drug users, the other main risk factor for hepatitis B [59]. The high hepatitis B virus infection prevalence among FSW in the CAR is an indication that active sexual transmission is an important factor in the spread of hepatitis B virus infection in the country. FSW may be a reservoir group for the maintenance and transmission of the hepatitis B virus, as previously hypothesized in Nigeria [60, 61]. Taken together, screening FSW for hepatitis B virus markers and vaccinating those who are negative would be worthwhile in the CAR, as recommended by WHO [62].

### Different FSW categories and HIV infection

Most previous studies on female sew work in Africa focused on women who were clearly identified as sex workers [17,63]. Other networks of female commercial sex in Africa have been poorly evaluated, as well as their association with HIV infection [45, 64, 65]. In the present study, both major types of sex work, *i*.*e*. professional and nonprofessional FSW, could be considered and analyzed, as previously reported from Burkina Faso by Nagot and colleagues [16]. We have shown that female sex work in the CAR is remarkably diverse and heterogeneous, as previously reported in the various contexts of commercial sex [66].

FSW of all categories are by them-selves vulnerable because of the unequal gender relationships, gender violence and by the criminalization of sex work in Africa [12]. Thus, the overall prevalence of HIV infection among study FSW was 19.1%, *i*.*e*. nearly 3-fold higher than among adult women in the general population living in the CAR [3]. This demonstrates clearly that FSWs in Bangui constitute a core group for HIV infection. By bivariate analysis, the variable “sex workers categories” was not significantly associated with HIV. Thus, HIV prevalence was similar among professional and nonprofessional FSW. Nevertheless, the prevalences of HIV infection varied considerably among different categories of sex workers. The differential risk for HIV infection according to the various types of sex work was previously reported in sub-Saharan Africa [16, 47]. Types of sexual partners, sexual activities, and socio-behavioral characteristics probably explain the large differences in the rates of HIV infection across categories.

The high rate of HIV infection presented by self-recognized professionals is consistent with the prevalences found in similar populations in other African countries. In Democratic Republic of Congo, Cameroon, Mali, Benin, Burkina Faso, and Ivory Coast, HIV prevalences found in self-identified sex workers were 20% [47], 24% [67], 30% [68], 53% [69], 57% [16], and 71% [70], respectively.

In our series, the lowest prevalence (<10%) was observed in the categories of “students” and "civil servants" among nonprofessional FSW and in the category of "*pupulenge*" among professional FSW. Indeed, “students” and "civil servants" are young, have a high education level and generally show good knowledge of HIV infection. These women negotiate part-time sexual services to supplement their often insufficient income. They therefore have fewer sexual partners, as previously reported [71]. The low prevalence of HIV among "*pupulengue*" may be likely explained by the fact that these FSW preferentially turn to rich, foreign and often White clients, thus selecting sexual partners with *a priori* lower risk for HIV infection. Furthermore, the categories of “students” and “*pupulenge*” consistently used condoms the most with their clients in more than 80% of sexual acts, likely explaining in part the low HIV prevalence in these two categories.

The category of “housewives” appeared highly vulnerable, with HIV prevalence of 13%. This unsuspected category of “clandestine” FSW is likely linked to the very difficult economical context of the CAR. Thus, some housewives or unemployed women have clearly insufficient income, finding no financial support from their parents or even their own spouse with whom they live. These women in extreme economic vulnerability multiply sexual relationship seeking material gains to solve their basic needs and for some of them their food needs. Women belonging to “housewives” category are particularly exposed to unsafe sex, because they are mostly unaware of the risk of HIV infection and are working "underground" without facilities to negotiate safe sex. Previous studies in sub-Saharan Africa have documented the association between low socio-economic status of FSW and high risk for HIV [72].

The category of professional “*kata*” and that of nonprofessional “street vendors” or “sellers” constituted the subgroups of FSW harboring the highest HIV prevalences (39.1% and 31.9%, respectively), thus much higher than among adult women in the general population living in the CAR [3]. The situation of “sellers” stresses the urgent need for adapted intervention. “Street vendors” are heavily infected, which is partly explained by their weak negotiation power for condom use. Their poor level of education and extremely low income (salary of 50€ per month on average) seem to be directly responsible for this situation. Moreover, according to our experience of the sex work network, the sexual partners of this category of FSW belong to high-risk groups. Indeed, “street vendors” are in contact with truck and taxi drivers, who now tend to engage in sex with nonprofessionals because they are aware the latter could be heavily infected.

The categories of “*kata*” and “street vendors”, although very different, have however common characteristics. Thus, they negotiate sexual services far from down town in the suburbs on the street, in neighborhoods, popular bars or dancing places. They are usually from very disadvantaged social backgrounds and are poorly educated. They are heavily affected by alcohol and other psycho-active substances, as previously reported [44]. In our experience, the small street trading in the CAR is frequently associated with sex work activity. Thus, most professional “*kata*” also once sold goods (peanut, coconut, salad or cassava) on the street. This observation implies that FSW may change category according to the economic context and possibilities. Finally, we estimate that only about 5% of women change categories over a period of 1 year.

Interestingly, the distribution of the various sex worker categories into professional and nonprofessional FSW for statistical analyses could not allow to differentiate factors associated with HIV between these two main FSW categories. This finding suggests that the professional *versus* nonprofessional categorization likely does not capture the different levels of risk for HIV infection, and thus does not appear as sufficiently operational for designing specific interventions.

### Possible interventions

Our study constitutes the starting point for a reflection on the management of FSW in the CAR, including health authorities, non-governmental organizations of people living with HIV and public health researchers. The identification of several categories of FSW will help to determine the hot spots of some forms of sex work across the country, and also to estimate the number of FSW to be targeted. Pilot interventions should be conceived, developed in existing health care facilities, such as STI clinics like the *CNRMST/SIDA* of Bangui, and thereafter evaluated. Targeted interventions that aim to increase condom use and reduce transmission of STIs and HIV infection among FSW and their clients have been shown to be feasible and effective [10, 73, 74]. Syndromic management of curable STIs is an essential part of interventions targeting FSW and their clients [75], but so far less attention has been paid to the diagnosis and management of bacterial vaginosis in FSW. More research needs to be done on how best to manage reproductive tract infections. In resource-limited settings, the syndromic approach is often recommended for the control of STIs in commercial sex networks [75]. However, the effectiveness of this approach may be problematic because most STIs are asymptomatic. This may change as new rapid diagnostic tests for STIs are becoming more and more accessible and affordable [76]. Concerning HSV-2 infection, however, recent clinical trials have not confirmed that suppressive treatment of herpetic infection would reduce the risk of HIV acquisition among women infected with HSV-2 as well as the risk of transmitting HIV to their partners in individuals co-infected with HIV and HSV-2 [77,78].

Population-specific minimum packages of services will be defined for each category of reachable FSW, including behavior change, communication and condom promotion by and in collaboration with peer educators, STIs screening and treatment, counselling and testing and care for the HIV infected. Controlling alcohol consumption must be integrated into prevention interventions among FSW. Indeed, reducing alcohol use among sex workers and their clients have led to a decrease in the number of unprotected sex acts, sexual violence, and finally of HIV and other STIs [41,79,80]. Combining biomedical approaches (oral pre-exposure prophylaxis together with HIV testing and antiretroviral treatment programs) with a prevention package, including behavioral and structural components as part of a community-driven approach, will help to reduce HIV infection in sex workers in sub-Saharan Africa [81].

Unexpectedly, YWSS were 27% of study FSW population. There are no accurate global estimates of the prevalence of children aged less than 18 years who sell sex. However, many studies show that substantial percentages of sex workers in many countries began selling while aged younger than 18 years. In Burkina Faso, 6% of FSW were less than 18 years in 2002 [16]. In eight countries in eastern and southern Africa, median HIV prevalence among sex workers younger than 25 years is 11% [82]. The YWSS group is more vulnerable than older cohorts to health harms—including STIs, HIV, and violence [82–84]. According to the Convention on the Rights of the Child—the most widely ratified human rights treaty [85], international agreements define YWSS under 18 years as victims of trafficking and/or sexual exploitation, and making many providers wary of legal repercussions under international laws for sexually exploited children. In our study, the recruitment was made on a voluntary basis, and we have no reason to believe that YWSS were constrained by pimps. Nonetheless, this vulnerable population needs specific health interventions including access to sexual and reproductive health and rights, and HIV treatment, prevention, and care, despite the law and policy barriers and the frequent lack of confidential and adolescent-friendly HIV services.

## Conclusion

Commercial female sex work is very heterogeneous in the Central African Republic involving “official” professional and “clandestine” nonprofessional FSW, with high and differential HIV prevalence among FSW. This suggests the need to design and develop programs against HIV and other STI adapted and specific to each FSW category.
